# Degree of joint risk factor control and premature mortality in hypertensive participants

**DOI:** 10.1093/pcmedi/pbaf006

**Published:** 2025-03-19

**Authors:** Jian Zhou, Minghao Kou, Rui Tang, Xuan Wang, Hao Ma, Xiang Li, Yoriko Heianza, Lu Qi

**Affiliations:** Department of Orthopedics, The Second Xiangya Hospital of Central South University, Changsha 410011, China; Department of Epidemiology, Tulane University School of Public Health and Tropical Medicine, New Orleans, LA 70112, USA; Postdoctoral Mobile Station of Clinical Medicine, The Second Xiangya Hospital of Central South University, Changsha, Hunan 410011, China; National Clinical Research Center for Metabolic Diseases, The Second Xiangya Hospital of Central South University, Changsha, China; Department of Epidemiology, Tulane University School of Public Health and Tropical Medicine, New Orleans, LA 70112, USA; Department of Epidemiology, Tulane University School of Public Health and Tropical Medicine, New Orleans, LA 70112, USA; Department of Epidemiology, Tulane University School of Public Health and Tropical Medicine, New Orleans, LA 70112, USA; Department of Epidemiology, Tulane University School of Public Health and Tropical Medicine, New Orleans, LA 70112, USA; Department of Epidemiology, Tulane University School of Public Health and Tropical Medicine, New Orleans, LA 70112, USA; Department of Epidemiology, Tulane University School of Public Health and Tropical Medicine, New Orleans, LA 70112, USA; Department of Epidemiology, Tulane University School of Public Health and Tropical Medicine, New Orleans, LA 70112, USA; Department of Nutrition, Harvard T.H. Chan School of Public Health, Boston, MA 02138, USA

**Keywords:** hypertensive participants, risk factor control, premature mortality, UK Biobank

## Abstract

**Objective:**

To investigate whether the excess premature mortality risk related to hypertension could be reduced or eliminated through joint risk factor control.

**Methods:**

A total of 70 898 hypertensive participants and 224 069 matched non-hypertensive participants without cancer or cardiovascular disease (CVD) at baseline were included and followed from 2006 to 2022. The degree of joint risk factor control was evaluated based on the major cardiovascular risk factors, including blood pressure, body mass index, waist circumference, low-density lipoprotein cholesterol, glycated haemoglobin, albuminuria, smoking, and physical activity. Cox proportional hazards models were used to investigate the relationship between degree of risk factor control and premature mortality.

**Results:**

Each additional risk factor control was associated with a 15%, 12%, 24%, and 11% lower risk of premature all-cause mortality, premature cancer mortality, premature CVD mortality, and premature other mortality, respectively. Optimal risk factor control (≥6 risk factors) was associated with a 55% [hazard ratio (HR): 0.45, 95% confidence interval (CI): 0.40–0.51], 50% (HR: 0.50, 95% CI: 0.41–0.60), 67% (HR: 0.33, 95% CI: 0.26–0.42), and 50% (HR: 0.50, 95% CI: 0.40–0.62) lower risk of premature all-cause mortality, premature cancer mortality, premature CVD mortality, and premature other mortality, respectively. Hypertensive participants with 3, 2, 4, and 2 or more controlled risk factors showed no excess risk of premature all-cause mortality, premature cancer mortality, premature CVD mortality, and premature other mortality, respectively, compared to matched non-hypertensive participants.

**Conclusions:**

In this cohort study of UK Biobank participants, degree of joint risk factor control shows gradient inverse association with risk of premature mortality in hypertensive participants; optimal risk factor control may eliminate hypertension-related excess risk of premature mortality.

## Introduction

Hypertension remains the leading risk factor for cardiovascular disease (CVD) and mortality [1]. Estimates from the 2010s indicate that hypertension impacted >30% of the adult population globally [2, 3]. Hypertension is a major cause of premature death worldwide and hypertensive participants carry a higher risk of premature mortality than the general population [4].

Recently, we observed a significant relationship between joint risk factor control and risk of heart failure in hypertensive participants; and optimal risk factor control eliminated hypertension-related excess risk of heart failure [5]. The latest evidence suggests that key cardiovascular risk factors, including blood pressure [6], smoking [7], cholesterol level [8], albuminuria [9], body mass index (BMI) [10], waist circumference [11], glycated haemoglobin [12], and physical activity [13], are related to risk of mortality in the general population. It has been found that jointly controlling multiple risk factors, including blood pressure, smoking, low-density lipoprotein cholesterol (LDL-C), glycated haemoglobin, and albuminuria, is related a lower risk of mortality in diabetes patients; and the diabetes-related excess risk of mortality is eliminated with all risk factors under control compared to non-diabetes controls [14, 15]. Currently, no study has tested the association between the degree of joint risk factor control and premature mortality in hypertensive participants; and no study has investigated whether the hypertension-related excess risk of premature mortality could be reduced or eliminated through joint risk factor control.

The objective of this study was to assess the associations between degree of joint risk factor control and risk of premature mortality in hypertensive participants. Additionally, we compared hypertensive participants with matched non-hypertensive participants to assess whether the hypertension-related excess premature mortality risk could be mitigated or nullified via jointly controlling risk factors.

## Methods

### Study population

The UK Biobank is a prospective cohort study collecting data from >500 000 community-dwelling adults aged 40–69 years from 2006 to 2010. Each participant provided comprehensive self-reported data via touchscreen questionnaires or oral interviews at UK Biobank's 27 assessment centres [16]. The study conducted by the UK Biobank received approval from the National Health and Social Care Information Management Board, the North West Multicenter Research Ethics Committee (11/NW/0382), and Tulane University's Institutional Review Board (2018–1872).

### Selection of participants

The hypertensive participants at baseline were defined based on International Classification of Diseases codes, 9th Revision (ICD 9) (codes: 401, 4010, 4011, 4019) or 10th revision (ICD 10) (code: I10), as well as a self-reported diagnosis by a physician [5] (supplementary Table 1, see online supplementary material). After excluding 45 participants who withdrew from the UK Biobank, 42 410 participants diagnosed with cancer, 29 003 participants diagnosed with CVD at baseline, 38 009 hypertensive participants with incomplete exposure data, and 184 unmatched participants, a total of 70 898 hypertensive participants remained. These hypertensive participants were then matched 1 : 4 with 224 069 non-hypertensive participants based on the following criteria: age within a 2-year difference, same gender, and same assessment centre (supplementary Fig. 1, see online supplementary material). The matching process was performed using a nearest-neighbour matching method with a maximum allowable age difference of 2 years, and exact matching on sex and assessment centre. A 1 : 4 matching ratio was applied, and matching was conducted with no transformation of variables. The final matched dataset includes hypertensive participants and their corresponding non-hypertensive controls.

### Definition of risk factors control

Eight risk factors were evaluated in our study, including blood pressure, BMI, waist circumference, LDL-C, glycated haemoglobin, albuminuria, smoking, and physical activity, with the number of controlled risk factors ranging from 0 to 8 (supplementary Table 2, see online supplementary material). Blood pressure was measured twice using an electronic blood pressure monitor (Omron 705 IT, OMRON Healthcare Europe B.V., Hoofddorp, The Netherlands) or a mercury sphygmomanometer by a trained nurse at the assessment centre. (i) Blood pressure control was defined as an average systolic blood pressure (SBP) < 140 mmHg and diastolic blood pressure (DBP) < 90 mmHg [17]. (ii) BMI was calculated as weight divided by height squared (kg/m²). A normal BMI, defined as 18.5 to <25 kg/m^2^ according to the World Health Organization (WHO) guidelines, was considered as BMI control. (iii) We categorized participants based on WHO thresholds for waist circumference [18]. Waist circumference ≤ 88 cm for women and ≤102 cm for men were considered as waist circumference control. (iv) Serum LDL-C was analyzed using the enzymatic selective protection technique (Beckman Coulter UK, Ltd). We recognized lipid control as LDL-C < 3.6 mmol/l [5]. (v) Glycated haemoglobin was quantified in a plasma sample gathered at baseline through high-performance liquid chromatography on a BioRad VARIANT II Turbo (Bio-Rad Laboratories, Inc). Glycated haemoglobin control was established as glycated haemoglobin < 48 mmol/mol [5]. (vi) Urinary microalbumin was ascertained using immunoturbidimetric assays (Randox Bioscience) with a detection threshold of 6.7 mg/l. Urine creatinine was analyzed by enzymatic methods (Beckman Coulter UK, Ltd). For those participants who displayed detectable microalbumin levels, the urinary albumin-to-creatinine ratio (uACR) was calculated by microalbumin and creatinine measurements. To include as many participants as possible, for those with undetectable microalbumin levels, the uACR was estimated by pairing an assumed microalbumin level (6.7 mg/l) and creatinine, with any uACR ≥ 3 mg/mmol recorded as missing value. Albuminuria was identified as an uACR ≥ 3 mg/mmol, while its absence was considered controlled albuminuria [5]. (vii) Information about smoking status was gathered via a touchscreen questionnaire, comprising categories for never, past, and current smoking. Non-current smokers were classified as smoking control. (viii) We defined the physical activity control as >150 min of moderate intensity activity per week, or >75 min of vigorous activity per week, or an equivalent combination per week [19].

### Outcomes

The main outcomes of our study included premature all-cause mortality, premature cancer mortality, premature CVD mortality, and premature other mortality. Premature mortality was defined as death occurring before the age of 80 years following previous study [20]. The final date for the follow-up was determined to be whichever of these three dates occurred first: the date of death, the censoring date (19 December 2022), or the date the individual reached 80 years of age. Mortality data was classified according to the International Classification of Diseases, 10th Revision (ICD 10). Specifically, our analysis included premature all-cause mortality, cancer premature mortality (ICD-10 codes C00-C97), CVD premature mortality (ICD-10 codes I00-I99), and other premature mortality [21].

### Covariates

Age, sex, ethnic background, Townsend deprivation index, education years, alcohol intake frequency, antihypertensive medication, hypertension duration, diabetes medication, and cholesterol-lowering medication were self-reported. Healthy diet score was calculated according to the consumption of vegetables, fruit, fish, processed meat, and unprocessed red meat (supplementary Table 3, see online supplementary material) following our previous study [22, 23]. We determined baseline diabetes by identifying participants with either a diabetes diagnosis before the date of attending the assessment centre, or those reporting a physician-diagnosed history of diabetes. For hypertension patients, we calculated the duration of hypertension from the diagnosis date to the study's baseline. For participants without hypertension, this duration was set as zero.

### Statistical analysis

We report continuous variables as means ± standard deviations and categorical variables as frequencies and percentages. Our study utilized Cox proportional hazards regression models to explore the relationship between the degree of joint risk factor control and the risk of premature mortality in hypertensive participants. To ensure the proportionality of hazards, we applied Schoenfeld residuals and Kaplan–Meier methods, meeting all predetermined criteria. In our analysis, hypertensive participants with the lowest risk factor control (≤2) were set as the reference group. The basic model was adjusted for age and sex, and the multivariable model was further adjusted for ethnic background, Townsend deprivation index, education years, alcohol intake frequency, healthy diet score, antihypertensive medication, hypertension duration, diabetes, diabetes medication, and cholesterol-lowering medication. The multivariable model was also used to compare the hypertensive participants and matched non-hypertensive participants. The missing data for categorical and continuous variables were imputed using a missing indicator category and mean imputation, respectively. The information for missing data of covariates is indicated in supplementary Table 4, see online supplementary material.

### Sensitivity analyses

We conducted two sensitivity analyses to verify the robustness of the findings. First, we deleted participants who died within the first 2 years of the follow-up period. Second, the participants with missing data for covariates were removed. We conducted all the statistical analyses using SAS version 9.4 (SAS Institute, Cary, NC) and R version 4.1.2 (www.r-project.org). A two-sided *P*-value < 0.05 was considered statistically significant.

## Results

### Baseline characteristics of participants

Table 1 indicates the baseline characteristics of included hypertensive participants and matched non-hypertensive participants. Among 70 898 hypertensive participants, 16.7%, 28.1%, 29.4%, 18.5%, and 7.3% had ≤3, 4, 5, 6, and ≥7 risk factors under control, respectively. In addition, we found that hypertensive participants with a higher degree of risk factor control tended to be older and predominantly female. They were more often older, female, White, possessed a higher socioeconomic status, received more years of education, and engaged in more frequent alcohol consumption. Their diets were generally healthier, and they were more likely to be taking cholesterol-lowering drugs, but less likely to be on antihypertensive medications and diabetes medication. These individuals typically had a longer duration of hypertension and were less likely to have diabetes.

**Table 1.** Baseline characteristics of included participants.

Characteristic	Non-hypertensive patients (*n* = 224 069)	Degree of joint risk factor control (*n* = 70 898)				
		≤3 Risk factors control (*n* = 11 849)	4 Risk factors control (*n* = 19 907)	5 Risk factors control (*n* = 20 829)	6 Risk factors control (*n* = 13 107)	≥7 Risk factors control (*n* = 5206)
Age, years, mean (SD)	57.4 (7.3)	57.6 (7.2)	58.5 (7.2)	58.9 (7.2)	59.3 (7.3)	58.7 (7.6)
Female, *n* (%)	114 936 (51.3)	5867 (49.5)	9403 (47.2)	8816 (42.3)	5660 (43.2)	2786 (53.5)
Ethnic background, *n* (%)						
Asian	7384 (3.3)	395 (3.3)	617 (3.1)	620 (3.0)	388 (3.0)	176 (3.4)
Black	1158 (0.5)	67 (0.6)	73 (0.4)	97 (0.5)	70 (0.5)	25 (0.5)
Chinese	694 (0.3)	8 (0.1)	40 (0.2)	54 (0.3)	55 (0.4)	28 (0.5)
Mixed	7581 (3.4)	457 (3.9)	756 (3.8)	729 (3.5)	431 (3.3)	169 (3.3)
White	204 104 (91.1)	10 767 (90.9)	18 209 (91.5)	19 122 (91.8)	12 024 (91.7)	4766 (91.6)
Other^a^	1778 (0.8)	116 (1.0)	155 (0.8)	145 (0.7)	98 (0.8)	29 (0.6)
Townsend deprivation index, mean (SD)	-1.5 (3.0)	-0.9 (3.2)	-1.2 (3.1)	-1.4 (3.0)	-1.6 (2.9)	-1.7 (2.9)
Education years, years, mean (SD)	15.1 (5.1)	14.4 (5.2)	14.5 (5.2)	14.8 (5.1)	15.0 (5.1)	15.3 (5.0)
Alcohol intake frequency, times/week, *n* (%)						
<3	121 052 (54.0)	6948 (58.6)	11 285 (56.7)	10 855 (52.1)	6514 (49.7)	2601 (50.0)
≥3	102 266 (45.6)	4895 (41.3)	8615 (43.3)	9968 (47.9)	6587 (50.3)	2602 (50.0)
Healthy diet score, *n* (%)						
<3	71 062 (31.7)	4808 (40.6)	7133 (35.8)	6659 (32.0)	3723 (28.4)	1238 (23.8)
≥3	143 678 (64.1)	6675 (56.3)	12 236 (61.5)	13 710 (65.8)	9085 (69.3)	3882 (74.6)
Antihypertensive medication, *n* (%)	2361 (1.1)	7791 (65.8)	13 265 (66.6)	13 582 (65.2)	8461 (64.6)	3262 (62.7)
Hypertension duration, years, *n* (%)						
<5	224 094 (100.0)	4443 (37.5)	7570 (38.0)	7928 (38.1)	4933 (37.6)	1910 (36.7)
5-<10	0 (0.0)	3156 (26.6)	5169 (26.0)	5489 (26.4)	3328 (25.4)	1345 (25.8)
≥10	0 (0.0)	3418 (28.9)	5869 (29.5)	6151 (29.5)	4086 (31.2)	1672 (32.1)
Diabetes, *n* (%)	5651 (2.5)	2439 (20.6)	2402 (12.1)	1728 (8.3)	748 (5.7)	208 (4.0)
Diabetes medication, *n* (%)	1171 (0.5)	546 (4.6)	404 (2.0)	264 (1.3)	110 (0.8)	34 (0.7)
Cholesterol-lowering medication, *n* (%)	17 793 (7.9)	3424 (28.9)	6135 (30.8)	6943 (33.3)	4469 (34.1)	1623 (31.2)

a UK Biobank did not define 'other' racial and ethnic groups.

### Degree of joint risk factor control and risk of premature mortality among hypertensive participants

**Figure 1.**
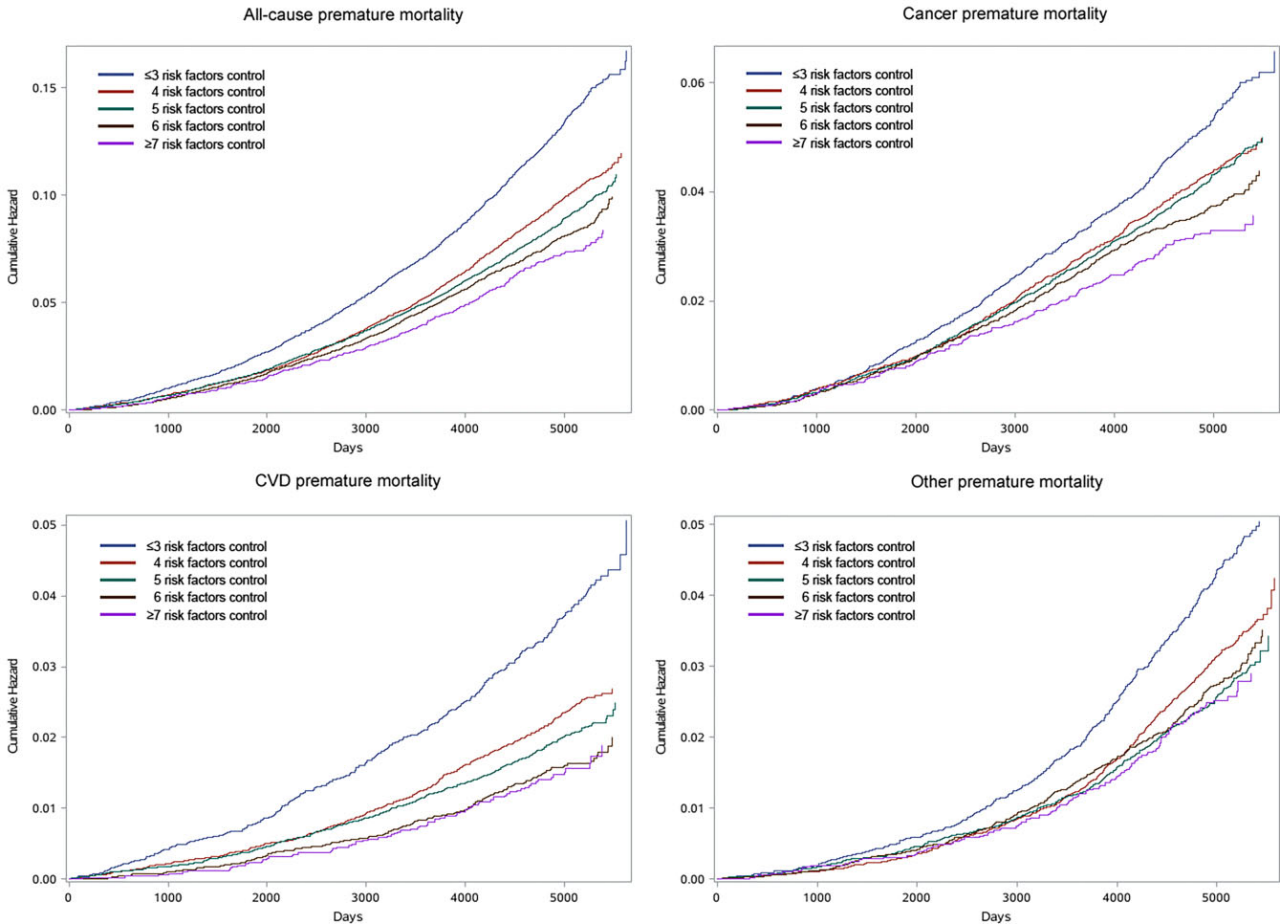
Cumulative hazard curves for the probability of premature mortality in hypertensive participants (*n* = 70 898) with varying degrees of joint risk factor control.

During a median follow-up period of 13.7 years, 6 466 documented premature deaths were recorded among 70 898 hypertensive participants, including 2 915 cancer premature deaths, 1 524 CVD premature deaths, and 2 027 other premature deaths. Figure 1 shows the cumulative hazard curves for premature mortality among hypertensive participants with varying degrees of joint risk factor control. We observed that the cumulative curves for hypertensive participants with lower risk factor control had a steeper gradient than matched non-hypertensive participants. In the basic model adjusted for age and sex, we observed that higher degree of joint risk factor control was significantly related to lower risks of premature mortality from all-cause, cancer, CVD, and other in hypertensive participants (Table 2). In the multivariable model, we found that each additional risk factor control was associated with a 13% lower risk of premature all-cause mortality [hazard ratio (HR): 0.87, 95% confidence interval (CI): 0.85–0.88], a 12% lower risk of premature cancer mortality (HR: 0.88, 95% CI: 0.86–0.91), a 21% lower risk of premature CVD mortality (HR: 0.79, 95% CI: 0.76–0.83), and a 10% lower risk of premature other mortality (HR: 0.90, 95% CI: 0.87–0.93) in hypertensive participants. The hypertensive participants with optimal risk factors control (≥7 risk factors) were associated with a 40% (HR: 0.60, 95% CI: 0.54–0.68), 39% (HR: 0.61, 95% CI: 0.51–0.73), 53% (HR: 0.47, 95% CI: 0.37–0.60), and 29% (HR: 0.71, 95% CI: 0.58–0.87) lower risk of premature all-cause mortality, premature cancer mortality, premature CVD mortality, and premature other mortality, respectively.

**Table 2.** Hazard ratios and 95% confidence intervals for association between degree of joint risk factor control and premature mortality in hypertensive patients (*n* = 70 898).

Outcome	Degree of joint risk factor control (*n* = 70 898)					Per 1 risk factor control
	≤3 Risk factors control (*n* = 11 849)	4 Risk factors control (*n* = 19 907)	5 Risk factors control (*n* = 20 829)	6 Risk factors control (*n* = 13 107)	≥7 Risk factors control (*n* = 5206)	
**All-cause premature mortality**						
Cases/person-years	1491/155 089	1853/262 642	1756/274 326	1006/172 567	360/68 815	
Incidence rate per 100 000 person-years	2.6 (2.5–2.8)	1.9 (1.8–2)	1.8 (1.7–1.8)	1.6 (1.5–1.7)	1.4 (1.3–1.6)	
Basic model^a^	1.00 (Reference)	0.68 (0.63–0.72)	0.58 (0.54–0.62)	0.51 (0.47–0.56)	0.49 (0.44–0.55)	0.82 (0.80–0.84)
Multivariable model^b^	1.00 (Reference)	0.74 (0.69–0.79)	0.67 (0.62–0.71)	0.61 (0.56–0.66)	0.60 (0.54–0.68)	0.87 (0.85–0.88)
**Cancer premature mortality**						
Cases/person-years	602/155 085	830/262 630	850/274 314	471/172 559	162/68 811	
Incidence rate per 100 000 person-years	1.1 (0.9818–1.2)	0.87 (0.81–0.93)	0.85 (0.79–0.91)	0.75 (0.68–0.82)	0.65 (0.55–0.75)	
Basic model	1.00 (Reference)	0.75 (0.68–0.84)	0.70 (0.63–0.78)	0.60 (0.53–0.68)	0.55 (0.46–0.65)	0.86 (0.83–0.89)
Multivariable model	1.00 (Reference)	0.78 (0.70–0.87)	0.75 (0.67–0.84)	0.66 (0.58–0.74)	0.61 (0.51–0.73)	0.88 (0.86–0.91)
**CVD premature mortality**						
Cases/person-years	418/155 084	440/262 630	395/274 305	196/172 557	75/68 812	
Incidence rate per 100 000 person-years	0.74 (0.67–0.81)	0.46 (0.42–0.50)	0.39 (0.36–0.44)	0.31 (0.27–0.36)	0.30 (0.24–0.37)	
Basic model	1.00 (Reference)	0.57 (0.50–0.65)	0.45 (0.40–0.52)	0.35 (0.29–0.41)	0.37 (0.29–0.47)	0.74 (0.71–0.77)
Multivariable model	1.00 (Reference)	0.64 (0.56–0.73)	0.54 (0.47–0.63)	0.43 (0.36–0.52)	0.47 (0.37–0.60)	0.79 (0.76–0.83)
**Other premature mortality**						
Cases/person-years	471/155 085	583/262 631	511/274 313	339/172 562	123/68 812	
Incidence rate per 100 000 person-years	0.83 (0.76–0.91)	0.61 (0.56–0.66)	0.51 (0.47–0.56)	0.54 (0.48–0.60)	0.49 (0.41–0.58)	
Basic model	1.00 (Reference)	0.68 (0.60–0.76)	0.54 (0.47–0.61)	0.55 (0.48–0.63)	0.54 (0.44–0.65)	0.83 (0.80–0.86)
Multivariable model	1.00 (Reference)	0.77 (0.68–0.87)	0.66 (0.58–0.75)	0.71 (0.61–0.82)	0.71 (0.58–0.87)	0.90 (0.87–0.93)

^a^Basic model: adjusted for age and sex. ^b^Multivariable model: adjusted for age, sex, ethnic background, Townsend deprivation index, education years, alcohol intake frequency, healthy diet score, antihypertensive medication, hypertension duration, diabetes, diabetes medication, and cholesterol-lowering medication.

### Degree of joint risk factor control and hypertension-related excess risk of premature mortality among hypertensive participants compared with matched non-hypertensive participants

**Figure 2.**
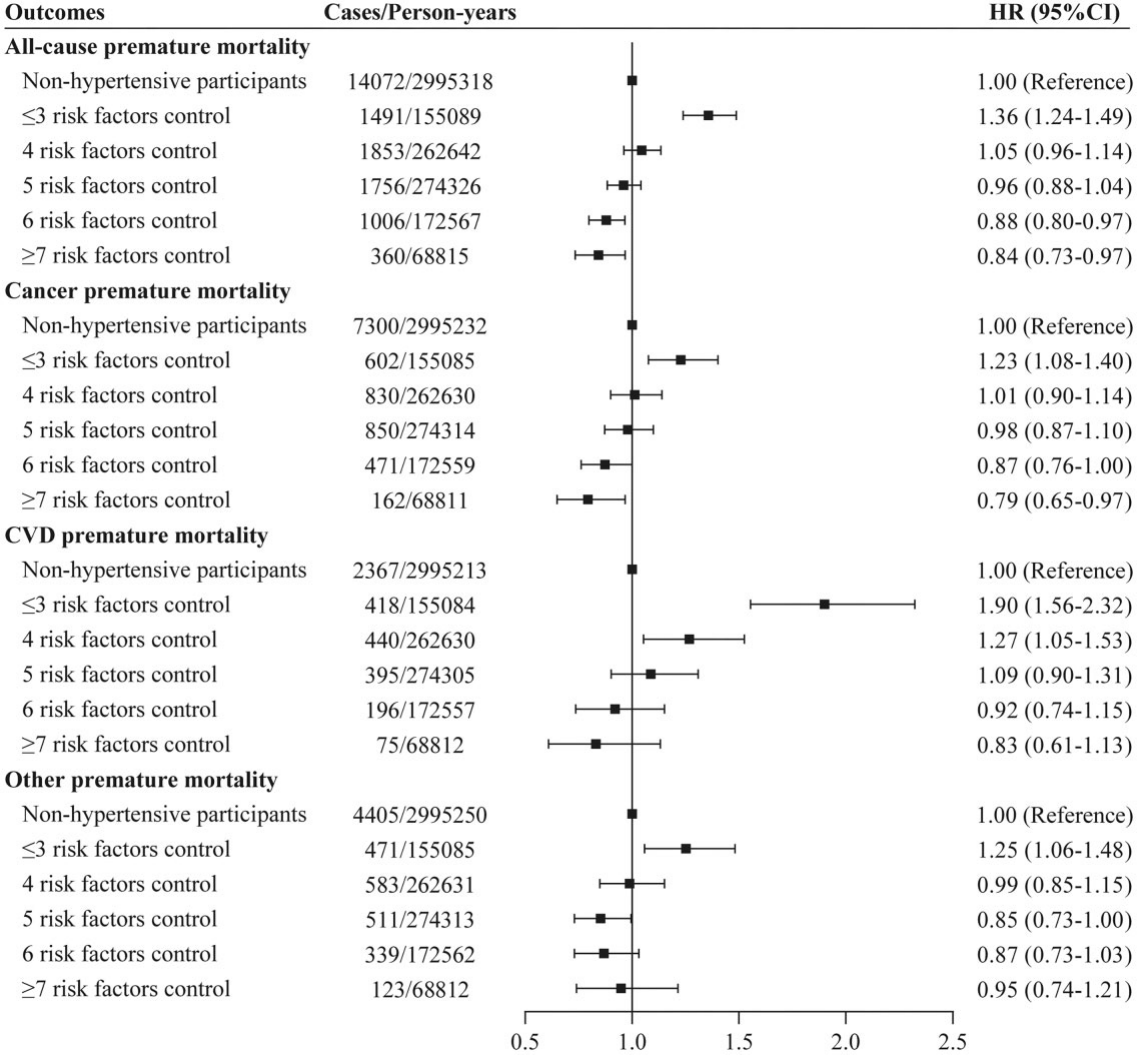
Association between degree of joint risk factor control and risk for premature mortality in hypertensive participants (*n* = 70 898) compared to matched non-hypertensive participants (*n* = 224 069) via the multivariable model. Multivariable model: adjusted for age, sex, ethnic background, Townsend deprivation index, education years, alcohol intake frequency, healthy diet score, antihypertensive medication, hypertension duration, diabetes, diabetes medication, and cholesterol-lowering medication.

During the follow-up, a total of 14 072 premature deaths were documented in the 224 069 matched non-hypertensive participants. As the degree of joint risk factor control increased, lower risks were observed for premature all-cause mortality, premature cancer mortality, and premature CVD mortality among hypertensive participants compared to matched non-hypertensive participants (Fig. 2). Hypertensive participants with 4, 4, 5, and ≥risk factors under control indicated no significant difference in risks of premature mortality compared to matched non-hypertensive participants. When the number of controlled risk factors increased from the lowest to the highest, the HR of premature all-cause mortality, premature cancer mortality, premature CVD mortality, and premature other mortality in hypertensive participants decreased by 52%, 44%, 107%, and 30%, respectively, compared with matched non-hypertensive participants (Fig. 2).

### Stratified analysis

**Figure 3.**
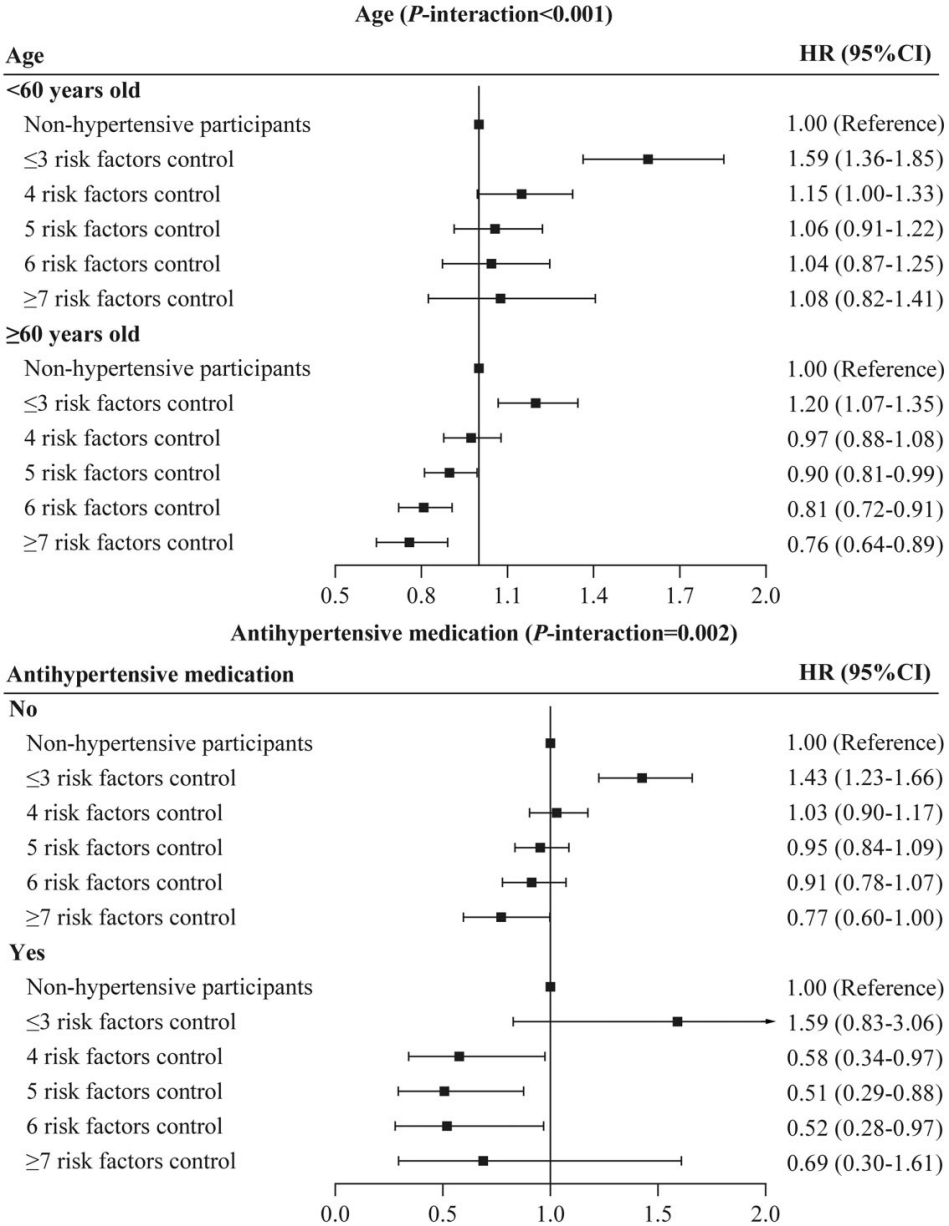
Stratified analysis for association between degree of joint risk factor control and risk for all-cause premature mortality in hypertensive participants (*n* = 70 898) compared with matched non-hypertensive participants (*n* = 224 069) via the multivariable model. Multivariable model: adjusted for age, sex, ethnic background, Townsend deprivation index, education years, alcohol intake frequency, healthy diet score, antihypertensive medication, hypertension duration, diabetes, diabetes medication, and cholesterol-lowering medication.

Figure 3 presents the results of stratified analyses according to age and antihypertensive medication. Lower risks were observed in hypertensive participants who were >60 years old (*P*-interaction < 0.001) and antihypertensive medication users (*P*-interaction = 0.034) with the same degree of risk factor control, as compared with matched non-hypertensive participants.

### Sensitivity analysis

Several sensitivity analyses were conducted to confirm the robustness of our findings. When we excluded premature deaths within the first 2 years of follow-up, our results remained consistent (supplementary Table 5 and supplementary Fig. 2, see online supplementary material). Similarly, our results remained unchanged after excluding the participants with missing data for covariates (supplementary Table 6 and supplementary Fig. 3, see online supplementary material).

## Discussion

In this prospective cohort study with a median follow-up of 13.7 years, we found that degree of joint risk factor control showed gradient inverse association with risk of premature mortality in hypertensive participants. Each additional risk factor control was associated with a 13%, 12%, 21%, and 10% lower risk of premature all-cause mortality, premature cancer mortality, premature CVD mortality, and premature other mortality, respectively. In addition, we found that optimal risk factor control (≥7 risk factors) was associated with a 40%, 39%, 53%, and 29% lower risk of premature all-cause mortality, premature cancer mortality, premature CVD mortality, and premature other mortality, respectively. Moreover, we found that hypertensive participants with 4, 4, 5, and ≥4 controlled risk factors, respectively, showed no excess risk of premature all-cause mortality, premature cancer mortality, premature CVD mortality, and premature other mortality, compared to matched non-hypertensive participants.

To our knowledge, this is the first study to explore the association between the degree of joint risk factor control and premature mortality in hypertensive participants. Intriguingly, we found that hypertension-related excess risk of premature mortality could be totally eliminated by joint risk factor control. Our findings are partly supported by two previous studies among diabetes patients, indicating that the diabetes-related excess risk of mortality decreased with increasing degrees of joint risk factor control, and optimal risk factor control could eliminate the diabetes-related excess risk of mortality [14, 15].

The mechanisms underlying the accumulative impact of joint risk factor control on lowering risk of premature remain to be clarified. Nevertheless, there are several possible mechanisms. Hypertension impacts numerous organs and bodily systems. CVD and chronic kidney diseases (CKD) emerge as principal complications in hypertensive participants [24], substantially increasing the risks of all-cause and premature CVD mortality. Effective management of different risk factors can potentially amplify the reduction of disease risk through distinct mechanisms. The synergistic control of multiple risk factors, including glycated haemoglobin, blood pressure, and LDL-C, can significantly diminish the risks of coronary artery disease and heart failure, thereby lowering the risk of all-cause and premature CVD mortality [25, 26]. Additionally, simultaneous control of blood pressure and albuminuria may reduce the risk of kidney failure, thereby reducing the premature mortality in hypertensive participants [27, 28]. Cardiorenal mechanisms play an important role in the development of multiple diseases and cardiorenal syndrome is related to adverse clinical outcomes [29, 30], underscoring the advantages of jointly managing CVD and CKD risk factors. Meanwhile, lifestyle-related factors including smoking, BMI, and physical activity are established risk factors for mortality [7,10,13]. Our findings indicated that joint lifestyle and cardiorenal risk factors control was associated with lower risk of premature mortality in hypertensive participants. Nevertheless, the mechanisms of joint risk factor control in reducing the risk of premature mortality remain to be further explored.

Our explorative study indicated that the HR of premature all-cause mortality, premature cancer mortality, premature CVD mortality, and premature other mortality in hypertensive participants decreased by 52%, 44%, 107%, and 30% respectively, as the number of controlled risk factors increased from the lowest to the highest, compared with matched non-hypertensive participants. Compared to controlling a single risk factor, multiple risk factors control presents more benefit in the reduction of premature mortality risk. Multiple risk factors control might have synergistic effects, reducing premature mortality risk through different mechanisms. Additionally, we noted that only 7.3% of hypertensive participants had >7 risk factors controlled, emphasizing the need to improve the management of risk factors in hypertensive participants.

It is notable that lower risks were observed in hypertensive participants who were >60 years old, with the same degree of risk factor control, as compared with matched non-hypertensive participants. Hypertensive participants >60 years old are more likely to be in poor health and often have other chronic diseases including type 2 diabetes and high cholesterol [31, 32]. Joint risk factor control might provide more benefit in hypertensive participants >60 years old. Additionally, we noted that the protective correlation was more pronounced in hypertensive participants who were antihypertensive medication users. Antihypertensive medication may enhance the effect of joint risk factor control on risk of mortality, thereby amplifying the impact of combined risk factor management. Our results suggest that hypertensive participants who were <60 years old and non-users of antihypertensive medication might show reduced responsiveness to joint risk factor control. This finding draws special attention to the suggestion that more intensive preventive approaches are warranted to achieve improved prevention of premature mortality.

Our study has several strengths, including its prospective design for a large cohort of hypertensive participants, and extensive details on covariates. However, it is important to acknowledge some limitations. First, we did not account for changes in variables related to risk factors that may have occurred during the follow-up period. Second, despite the thorough adjustment for various covariates, the possibility of residual confounding cannot be ruled out. Third, our study primarily involved White Europeans, highlighting the need for additional research to assess whether these results are applicable to other ethnic and racial groups. Fourth, the observational design of this study limits our ability to obtain causal relationships. Finally, due to data limitations and incomplete reporting of lifestyle factors across participants, we did not include them in our analysis.

## Conclusions

Degree of joint risk factor control shows gradient inverse association with risk of premature mortality in hypertensive participants. Importantly, our findings indicate that optimal risk factor control may eliminate the hypertension-related excess risk of premature mortality, emphasizing the importance of joint risk factor control in the prevention of premature mortality in hypertensive participants.

## Supplementary Material

pbaf006_Supplemental_File

## Data Availability

This study was conducted using the UK Biobank Resource, approved project number 29256. The UK Biobank will make the source data available to all bona fide researchers for all types of health-related research that is in the public interest, without preferential or exclusive access for any persons. All researchers will be subject to the same application process and approval criteria as specified by UK Biobank. For more details on the access procedure, see the UK Biobank website: https://www.ukbiobank.ac.uk.
