# Supplementary material for: Degree of joint risk factor control and premature mortality in hypertensive participants
Source: Precis Clin Med. 2025 Mar 19;8(2):pbaf006. doi: 10.1093/pcmedi/pbaf006 (PMC11979693; doi:10.1093/pcmedi/pbaf006)
Supplement: pbaf006_Supplemental_File [file pbaf006_supplemental_file.docx]

**Supplementary Data**

**Supplementary Figure 1.** Flow chart.

**Supplementary Figure 2.** Association between degree of joint risk factor control and premature mortality in hypertensive patients (n=70,602) compared with matched non-hypertensive patients (n=223,439) after excluding participants who were dead during the first two years of follow-up via multivariable model.

**Supplementary Figure 3.** Association between degree of joint risk factor control and premature mortality in hypertensive patients (n=64,181) compared with matched non-hypertensive patients (n=211,375) after excluding participants with missing covariate data via multivariable model.

**Supplementary Table 1.** Definitions of hypertensive patient.

**Supplementary Table 2.** Evaluation of risk factors in the UK Biobank.

**Supplementary Table 3.** Assessment of healthy diet score in the UK Biobank.

**Supplementary Table 4.** The numbers and percentages of participants with missing covariates.

**Supplementary Table 5.** Association between degree of joint risk factor control and premature mortality in hypertensive patients (n=70,898) after excluding participants who were dead during the first two years of follow-up via multivariable model.

**Supplementary Table 6.** Association between degree of joint risk factor control and premature mortality in hypertensive patients (n=64,181) after excluding participants with missing covariate data via multivariable model.Supplement Table 2. Evaluation of risk factors in the UK Biobank.

**Supplementary Table 3.** Assessment of healthy diet score in the UK Biobank.

**Supplementary Table 4.** The numbers and percentages of participants with missing covariates.

**Supplementary Table 5.** Association between degree of joint risk factor control and premature mortality in hypertensive patients (n=70,898) after excluding participants who were dead during the first two years of follow-up via multivariable model.

**Supplementary Table 6.** Association between degree of joint risk factor control and premature mortality in hypertensive patients (n=64,181) after excluding participants with missing covariate data via multivariable model.


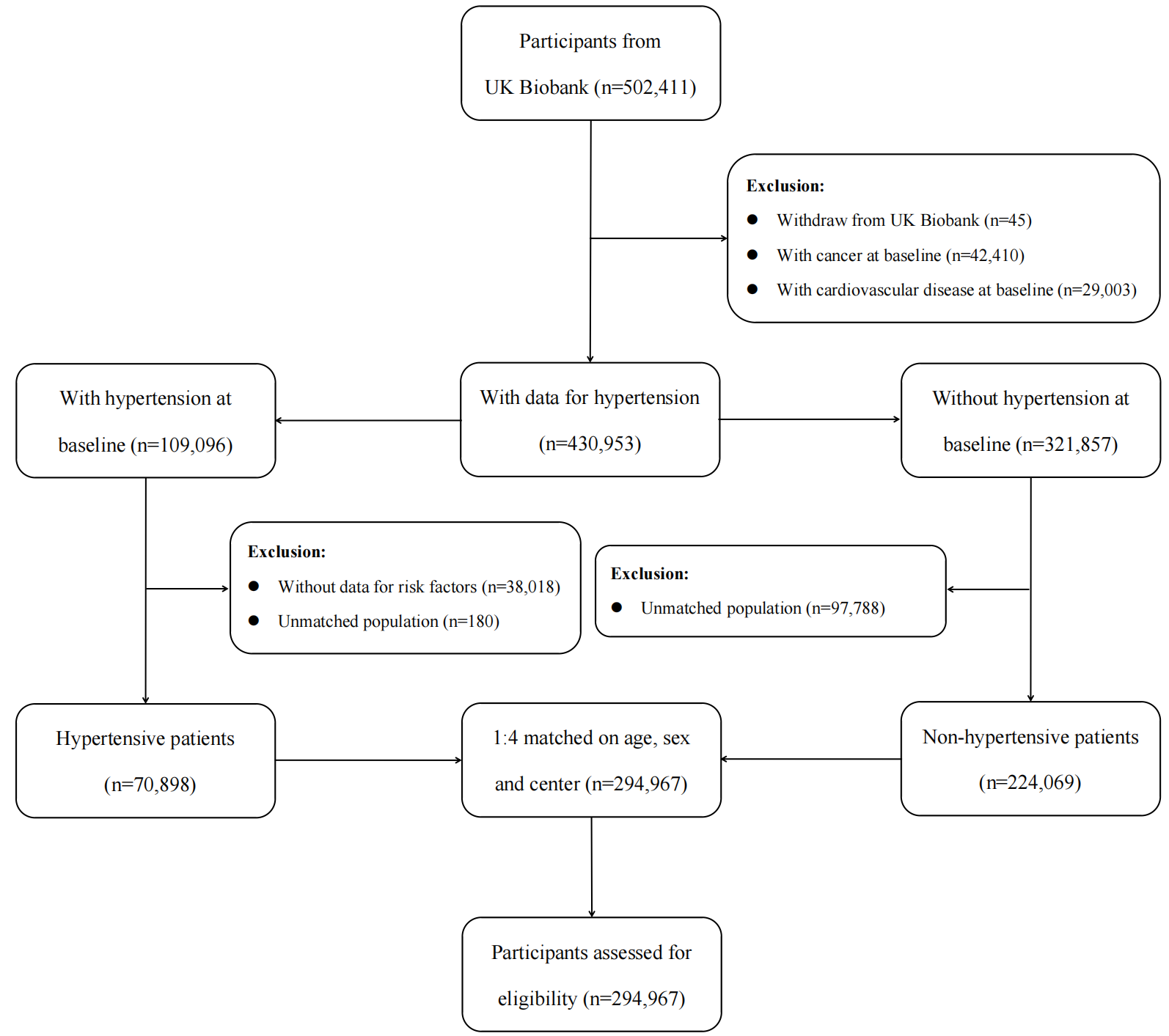


**Supplementary Figure 1.** Flow chart.


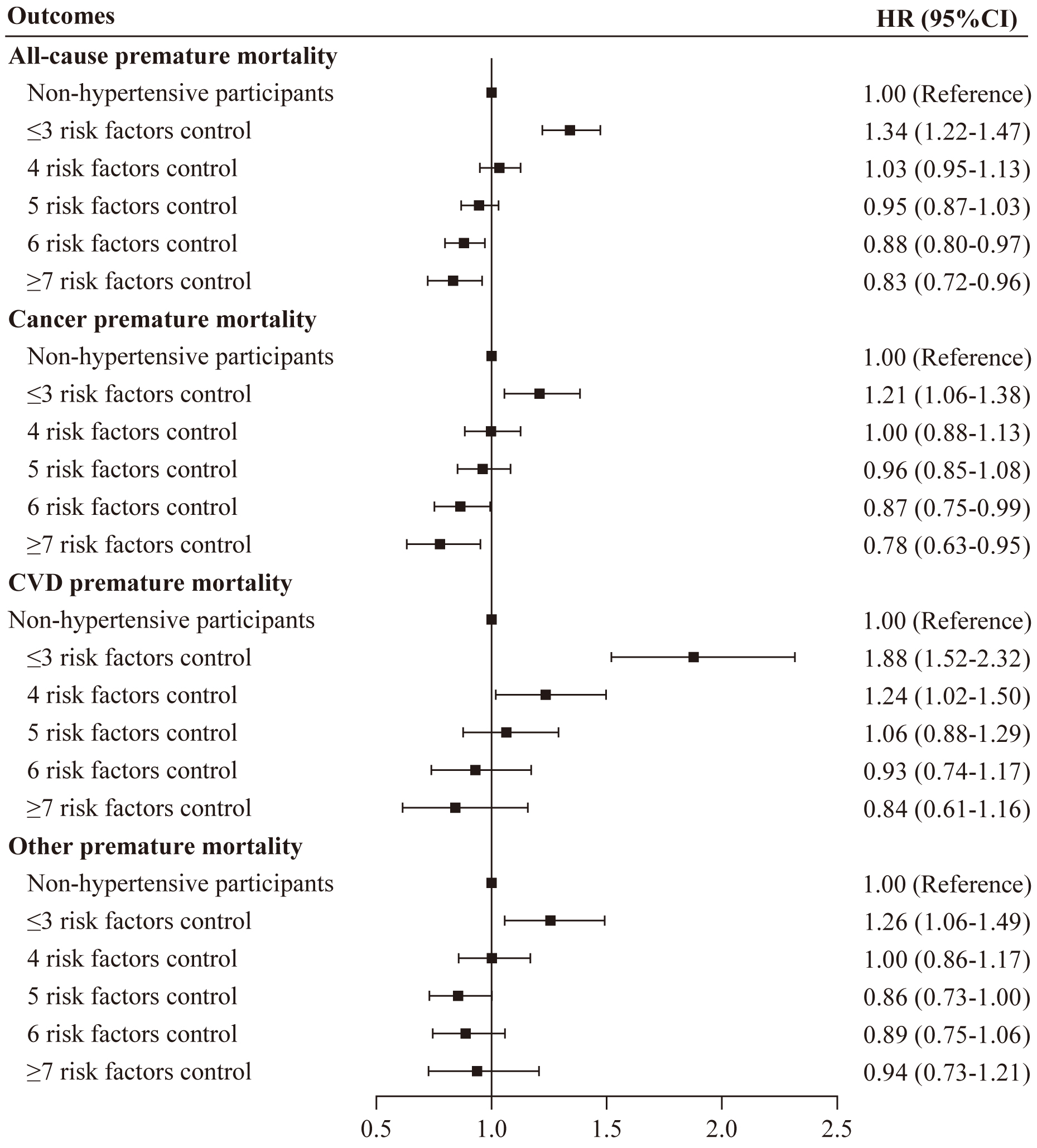


**Supplementary Figure 2.** Association between degree of joint risk factor control and premature mortality in hypertensive patients (n=70,602) compared with matched non-hypertensive patients (n=223,439) after excluding participants who were dead during the first two years of follow-up via multivariable model. CVD: cardiovascular disease. Multivariable model: adjusted for age, sex, ethnic background, Townsend deprivation index, education years, alcohol intake frequency, healthy diet score, antihypertensive medication, hypertension duration, diabetes, diabetes medication and cholesterol-lowering medication.

**
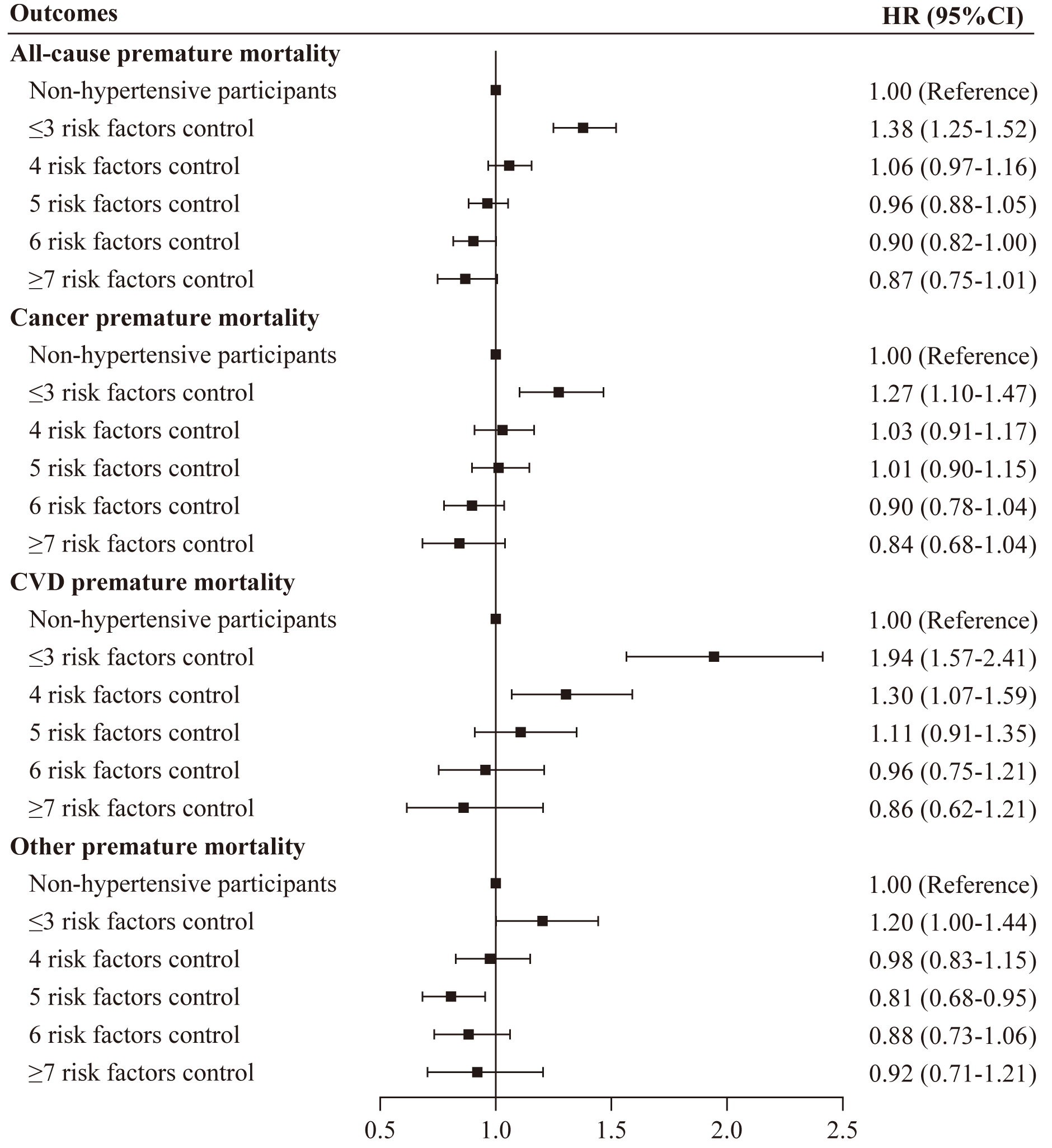
Supplementary Figure 3.** Association between degree of joint risk factor control and premature mortality in hypertensive patients (n=64,181) compared with matched non-hypertensive patients (n=211,375) after excluding participants with missing covariate data via multivariable model. CVD: cardiovascular disease. Multivariable model: adjusted for age, sex, ethnic background, Townsend deprivation index, education years, alcohol intake frequency, healthy diet score, antihypertensive medication, hypertension duration, diabetes, diabetes medication and cholesterol-lowering medication.

**Supplementary Table 1.** Definitions of hypertensive patient.

|  | **UK Biobank ID** | **Code or definition** ^1^ |
| --- | --- | --- |
| ICD 10 | 41270, 41280 | I10 |
| ICD 9 | 41271, 41281 | 401,4010,4011,4019 |
| Diagnosed by doctor | 6150, 2966 | 4 |

^1^ Kou M, Wang X, Ma H, Li X, Heianza Y, Qi L. Degree of Joint Risk Factor Control and Incident Heart Failure in Hypertensive Patients. JACC Heart Fail. 2023 Jun;11(6):678-688.

**Supplementary Table 2.** Evaluation of risk factors in the UK Biobank.

| **Risk factors** | **UK Biobank field ID** | **Description** | **Risk factor control** |
| --- | --- | --- | --- |
| Blood pressure | 93 | Systolic blood pressure, manual reading | Systolic blood pressure<130 mmHg and diastolic blood pressure <80 mmHg |
|  | 94 | Diastolic blood pressure, manual reading |  |
|  | 4079 | Diastolic blood pressure, automated reading |  |
|  | 4080 | Systolic blood pressure, automated reading |  |
| BMI | 21001 | Body mass index (BMI) | 18.5≤BMI<25 kg/m^2^ |
| Waist circumference | 48 | Waist circumference for women | <88 cm |
|  |  | Waist circumference for men | <102 cm |
| LDL cholesterol | 30780 | LDL direct | LDL cholesterol<2.5mmol/L |
| Glycated haemoglobin | 30750 | Glycated haemoglobin (HbA1c) | Glycated haemoglobin<53mmol/mol |
| Albuminuria | 30505 | Microalbumin in urine result flag | Microalbumin/Creatinine<3mg/mmol |
|  | 30510 | Creatinine (enzymatic) in urine |  |
| Smoking | 20116 | Smoking status | Non-current smoker |
| Physical activity | 884 | [Number of days/week of moderate physical activity 10+ minutes](https://biobank.ndph.ox.ac.uk/showcase/field.cgi?id=884) | ≥150 minutes per week |
|  | 894 | Duration of moderate activity |  |
|  | 904 | Number of days/week of vigorous physical activity 10+ minutes | ≥75 minutes per week |
|  | 914 | Duration of vigorous activity |  |

**Supplementary Table 3**. Assessment of healthy diet score in the UK Biobank.

| **Diet** | **UK Biobank field ID** | **Description** | **Healthy diet score** |
| --- | --- | --- | --- |
| Vegetable | 1289 | Cooked vegetable intake | 1 for ≥4 tablespoons/day  0 for <4 tablespoons/day |
|  | 1299 | Salad/raw vegetable intake |  |
| Fruit | 1309 | Fresh fruit intake | 1 for ≥3 pieces/day  0 for <3 pieces/day |
|  | 1319 | Dried fruit intake |  |
| Fish | 1329 | Oily fish intake | 1 for ≥2 times/week  0 for <2 times/week |
|  | 1339 | Non-oily fish intake |  |
| Processed meat | 1349 | Processed meat intake | 1 for ≤2 times/week  0 for >2 times/week |
| Unprocessed red meat | 1369 | Beef intake | 1 for ≤2 times/week  0 for >2 times/week |
|  | 1379 | Lamb/mutton intake |  |
|  | 1389 | Pork intake |  |

**Supplementary Table 4**. The numbers and percentages of participants with missing covariates.

| **Variable** | **N** | **%** |
| --- | --- | --- |
| Healthy diet score | 11078 | 3.76 |
| Hypertension duration | 4431 | 1.50 |
| Education years | 3303 | 1.12 |
| Antihypertensive medication | 2686 | 0.91 |
| Diabetes medication | 2686 | 0.91 |
| Cholesterol-lowering medication | 2686 | 0.91 |
| Ethnic background | 1582 | 0.54 |
| Alcohol intake frequency | 779 | 0.26 |
| Townsend deprivation index | 336 | 0.11 |

**Supplementary Table 5**. Association between degree of joint risk factor control and premature mortality in hypertensive patients (n=70,898) after excluding participants who were dead during the first two years of follow-up via multivariable model.

| **Outcomes** | **≤2 risk factor** | **3 risk factors** | **4 risk factors** | **5 risk factors** | **≥6 risk factors** | **Per 1 risk factor control** |
| --- | --- | --- | --- | --- | --- | --- |
| All-cause premature mortality | 1.00 (Reference) | 0.74 (0.69-0.79) | 0.66 (0.62-0.71) | 0.61 (0.56-0.67) | 0.60 (0.54-0.68) | 0.87 (0.85-0.89) |
| Cancer premature mortality | 1.00 (Reference) | 0.78 (0.70-0.87) | 0.75 (0.67-0.83) | 0.65 (0.58-0.74) | 0.60 (0.50-0.72) | 0.89 (0.86-0.92) |
| CVD premature mortality | 1.00 (Reference) | 0.64 (0.56-0.74) | 0.55 (0.47-0.63) | 0.44 (0.37-0.53) | 0.49 (0.38-0.63) | 0.80 (0.76-0.84) |
| Other premature mortality | 1.00 (Reference) | 0.77 (0.68-0.87) | 0.65 (0.57-0.74) | 0.71 (0.61-0.82) | 0.71 (0.57-0.87) | 0.90 (0.87-0.94) |

CVD: cardiovascular disease. Multivariable model: adjusted for age, sex, ethnic background, Townsend deprivation index, education years, alcohol intake frequency, healthy diet score, antihypertensive medication, hypertension duration, diabetes, diabetes medication and cholesterol-lowering medication.

**Supplementary Table 6**. Association between degree of joint risk factor control and premature mortality in hypertensive patients (n=64,181) after excluding participants with missing covariate data via multivariable model.

| **Outcomes** | **≤2 risk factor** | **3 risk factors** | **4 risk factors** | **5 risk factors** | **≥6 risk factors** | **Per 1 risk factor control** |
| --- | --- | --- | --- | --- | --- | --- |
| All-cause premature mortality | 1.00 (Reference) | 0.73 (0.68-0.79) | 0.66 (0.62-0.72) | 0.61 (0.56-0.66) | 0.59 (0.52-0.67) | 0.87 (0.85-0.89) |
| Cancer premature mortality | 1.00 (Reference) | 0.78 (0.69-0.87) | 0.75 (0.67-0.84) | 0.64 (0.56-0.74) | 0.61 (0.51-0.74) | 0.89 (0.86-0.92) |
| CVD premature mortality | 1.00 (Reference) | 0.62 (0.54-0.72) | 0.54 (0.47-0.63) | 0.44 (0.36-0.52) | 0.44 (0.34-0.58) | 0.79 (0.75-0.83) |
| Other premature mortality | 1.00 (Reference) | 0.77 (0.68-0.88) | 0.65 (0.56-0.74) | 0.72 (0.62-0.84) | 0.70 (0.56-0.86) | 0.90 (0.87-0.94) |

CVD: cardiovascular disease. Multivariable model: adjusted for age, sex, ethnic background, Townsend deprivation index, education years, alcohol intake frequency, healthy diet score, antihypertensive medication, hypertension duration, diabetes, diabetes medication and cholesterol-lowering medication.
